# miR-29a Participated in Nacre Formation and Immune Response by Targeting *Y2R* in *Pinctada martensii*

**DOI:** 10.3390/ijms161226182

**Published:** 2015-12-10

**Authors:** Rongrong Tian, Zhe Zheng, Ronglian Huang, Yu Jiao, Xiaodong Du

**Affiliations:** 1 Fishery College, Guangdong Ocean University, Zhanjiang 524025, China; Rongrong_Tian619@126.com (R.T.); haidazhengzhe@163.com (Z.Z.); hrl8849@163.com (R.H.); 2 Guangdong Technology Research Center for Pearl Aquaculture and Process, Guangdong Ocean University, Zhanjiang 524025, China

**Keywords:** Pm-miR-29a, *Pinctada martensii*, nacre formation, biomineralization, immune response

## Abstract

miR-29a is a conserved miRNA that participates in bone formation and immune response in vertebrates. miR-29a of *Pinctada martensii* (Pm-miR-29a) was identified in the previous research though deep sequencing. In this report, the precise sequence of mature Pm-miR-29a was validated using miRNA rapid amplification of cDNA ends (miR-RACE) technology. The precursor sequence of Pm-miR-29a was predicted to have 87 bp. Stem loop qRT-PCR analysis showed that Pm-miR-29a was easily detected in all the tissues, although expressions in the mantle and gill were low. The microstructure showed the disrupted growth of the nacre after Pm-miR-29a over-expression, which was induced by mimic injection into *P. martensii*. Results of the target analysis indicated that neuropeptide Y receptor type 2 (*Y2R*) was the potential target of Pm-miR-29a. Meanwhile, Pm-miR-29a mimics could obviously inhibit the relative luciferase activity of the reporter containing 3′ UTR (Untranslated Regions) of the *Y2R* gene. Furthermore, the expression of *Y2R* was downregulated whereas expressions of interleukin 17 (*IL-17*) and nuclear factor κB (*NF-κB*) were upregulated after Pm-miR-29a over-expression in the mantle and gill, thereby suggesting that Pm-miR-29a could activate the immune response of the pearl oyster. Results showed that Pm-miR-29a was involved in nacre formation and immune response by regulating *Y2R* in pearl oyster *P. martensii*.

## 1. Introduction

MicroRNAs (miRNAs) are a kind of endogenous non-coding small single-stranded RNA (18–25 nucleotides). In animals, miRNA is initially transcribed to form a longer primary miRNA. In the nucleus, the miRNA is processed into a hairpin precursor miRNA by the Drosha, and then finally cut into double-stranded mature miRNA of about 22 nucleotides by the Dicer enzyme in the cytoplasm. The mature miRNA could bind with the 3′ UTR of target mRNA for complete or incomplete complementary pairing, which results in the degradation or translational repression of mRNA, thereby affecting expression of the target genes [1]. Increasing evidence has shown that miRNA participates in nearly all cellular processes, such as cell proliferation, differentiation, apoptosis, and immunity, by targeting various genes [2,3,4]. Reports showed that miRNAs could regulate about 30% of protein-coding genes in humans [5]. Meanwhile, the miR-29 family, containing miR-29a, miR-29b, and miR-29c, is one of the most widely investigated regulators of the extracellular matrix, such as osteonectin [6] and collagen [7]. Researchers have collectively shown that miR-29a positively regulated osteoblast differentiation and protected bone from fibrosis and/or restricted collagen accumulation by targeting osteonectin [6] and collagen [7,8,9].

In our previous research, miR-29a was found in the pearl oyster *Pinctada martensii* (Pm-miR-29a) [10], although its function in the pearl oyster is still unclear. Considering the homology between bone and shell formations, we proposed that miR-29a is possibly involved in shell formation in the pearl oyster. This study aimed to investigate the exact functions of Pm-miR-29a in the pearl oyster *P. martensii* to elucidate the intricate and extensive role of this miRNA family.

## 2. Results

### 2.1. Sequence Verification of Mature Pm (Pinctada martensii)-miR-29a

**Figure 1:**
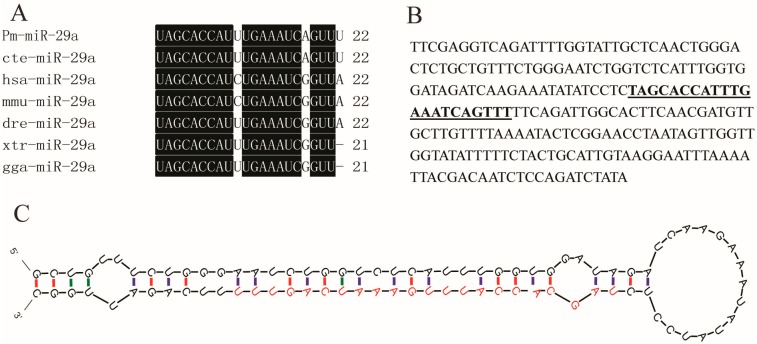
Sequence analysis of Pm-miR-29a. (**A**) Multi-alignment of mature miR-29a of *Pinctada martensii* (Pm-miR-29a) with that from other species (cte: Capitella teleta; hsa: Homo sapiens; mmu: Mus musculus; dre: Danio rerio; xtr: Xenopus tropicalis; gga: Gallus gallus). The conserved nucleotides in all animals were written in white on a black background; (**B**) Nucleotides surrounding the mature miR-29a, the bold style and underlined nucleotides represented the mature Pm-miR-29a; (**C**) Secondary structure of the precusor Pm-miR-29a analyzed by M-fold, the red nucleotides represented the mature Pm-miR-29a, the green and purple colors respectively means G-U cannot complementary pair but A-U base pair.

miR-RACE is an efficient method to validate the precise sequences of mature miRNAs; it has successfully been used to validate the precise miRNA sequences in some species [11]. First, 5′ and 3′ miR-RACE was conducted to validate the sequence of mature Pm-miR-29a obtained by Solexa deep sequencing. Figure S1 shows that both 5′ and 3′ Pm-miR-29a sequences were consistent with those obtained by Solexa deep sequencing. Multi-alignment of the mature Pm-miR-29a with that from other species showed that all analyzed miRNAs shared the same nucleotide sequence at the seed region. However, the 10th cytosine of Pm-miR-29a was replaced by uracil, similar with that in dre-miR-29a from *Danio rerio* and cte-miR-29a from *Capitella teleta*. In *P. martensii* and *C. teleta*, the eighth guanine and the 21st adenine were changed into adenine and uracil, respectively (Figure 1A). In addition, based on the reported genome sequence [12], we obtained the nucleotides surrounding the mature miR-29a (Figure 1B). The secondary structure analysis by M-fold software indicated that mature Pm-miR-29a was contained in one characteristic hairpin structure with 87 bp, which was predicted as the precursor of Pm-miR-29a (Figure 1C). Hence, we further verified the precise sequence and existence of miR-29a in *P. martensii*.

### 2.2. Expression and Distribution of Pm-miR-29a in Different Tissues

**Figure 2:**
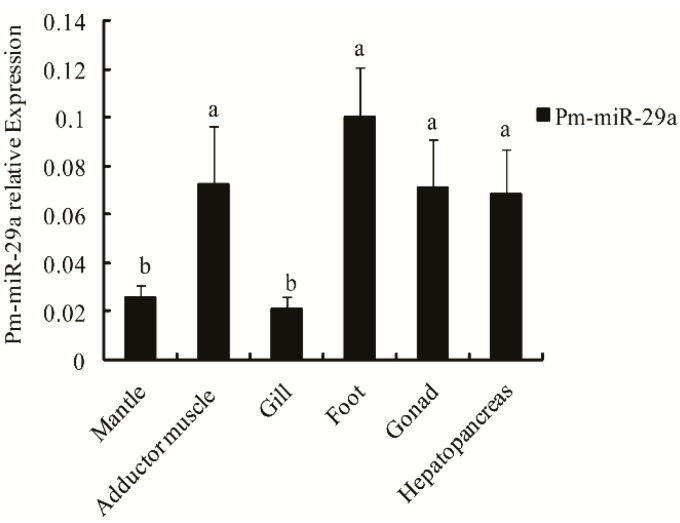
Expression levels of Pm-miR-29a in different tissues. qRT-PCR was done with RNA samples from the adductor muscle, gill, mantle, hepatopancreas, gonad, foot. *U6 snRNA* was used as the internal reference gene. Different letters mean a significant difference in all detected tissues (*p* < 0.05). Error bars correspond to mean ± SD.

To further prove the existence of miR-29a in *P. martensii*, stem loop qRT-PCR analysis, with *U6 snRNA* as the reference gene, was applied to detect the Pm-miR-29a expression profile in different tissues, including the mantle, adductor muscle, gill, foot, gonad and hepatopancreas. Results showed that Pm-miR-29a was easily detected in all tissues with high expressions in the foot, adductor muscle, gonad, and hepatopancreas, and low expressions in the mantle and gill (Figure 2).

### 2.3. Functions of Pm-miR-29a in Nacreous Layer Formation

**Figure 3:**
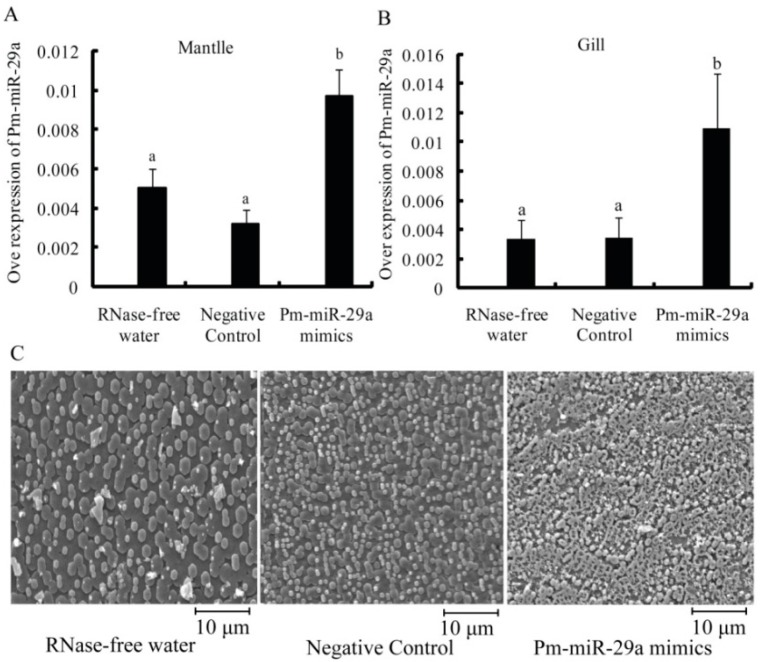
Functional analysis of Pm-miR-29a.The over-expression of Pm-miR-29a in the mantle (**A**) or gill (**B**) after Pm-miR-29a mimic injection were all detected by stem loop qRT-PCR. *U6 snRNA* was used as the internal reference gene. Different letters mean a significant difference (*p* < 0.05). Error bars correspond to mean ± SD; and (**C**) scanning electron microscope (SEM) images of the nacre after Pm-miR-29a mimic injection. The bars are 10 μm in the nacre images.

To verify the functional mechanism of Pm-miR-29a in *P. martensii*, Pm-miR-29a mimics were injected into the adductor muscle of *P. martensii*. Stem loop qRT-PCR was used to measure the expression levels of Pm-miR-29a in the mantle and gill at 8 d after the first injection. As seen in Figure 3A, the expression level of Pm-miR-29a was increased 2.35-fold in mantle and 3.22-fold in gill (Figure 3B) compared with the control group, which indicated that Pm-miR-29a mimics were successfully injected and the over-expression of Pm-miR-29a *in vivo* was induced.

Meanwhile, scanning electron microscope (SEM) results revealed the microstructure of nacre 8 d after the first injection in each group. As shown in Figure 3C, the nacre showed a normal growth status in control groups. Many free aragonite crystals with smooth surfaces were closely packed, and the boundaries were clear. However, a honeycomb growth with a rough surface was observed in the Pm-miR-29a mimic-injected group. This result indicated that the over-expression of Pm-miR-29a affected the growth status of the crystal in the nacre.

### 2.4. Target Prediction

The next step was to find the potential target genes of Pm-miR-29a. During target analysis, only the reported genes in *P. martensii* with 3′ UTR were selected. Then, based on the results of gene annotation and target prediction by RNAhybrid software (Figure S2), the neuropeptide Y receptor type 2 (*Y2R*), fibroblast growth factor-18 (*FGF18*) and protein serine kinase (*SK*) were selected as the target genes of Pm-miR-29a.

### 2.5. Target Verification

**Figure 4:**
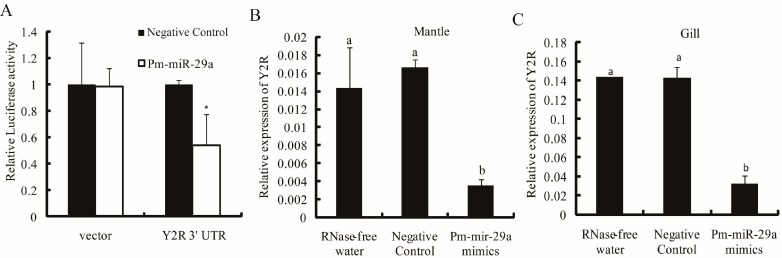
Target verification between Pm-miR-29a and *Y2R*. (**A**) Pm-miR-29a mimics obviously inhibited the luciferase activity of the 3′ UTR of the *Y2R* gene detected by dual-luciferase analysis. “*” represents a significant difference (*p* < 0.05). The relative expression of *Y2R* in the mantle (**B**) and gill (**C**) were detected by qRT-PCR after Pm-miR-29a mimic injection. *GAPDH* gene was used as the reference gene. Different letters mean a significant difference (*p* < 0.05); error bars correspond to mean ± SD.

**Figure 5:**
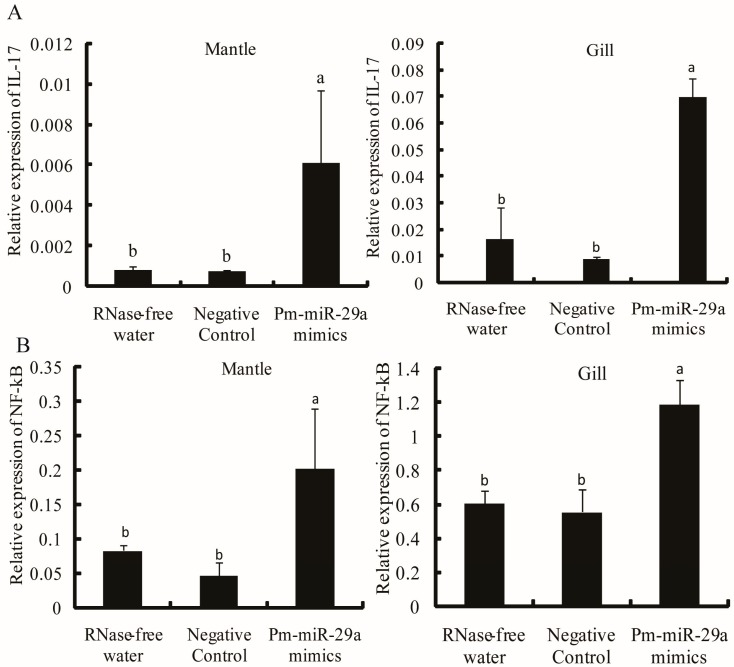
Expression level of the *IL-17* and *NF-κB* genes after over-expression of Pm-miR-29a. The expression level of *IL-17* (**A**) and *NF-κB* (**B**) in the mantle and gill were detected by qRT-PCR. *GAPDH* gene was used as the internal control. Different letters mean a significant difference (*p* < 0.05); error bars correspond to mean ± SD.

To demonstrate that *Y2R*, *FGF18* and *SK* were negatively regulated by Pm-miR-29a, we performed dual luciferase report analysis using luciferase reporters containing 3′ UTR of the *Y2R*, *FGF18* and *SK*. Pm-miR-29a mimics or the negative control were transfected into HEK-293T cells with the reporter plasmids. The cells were subjected to luciferase assays after 24 h of incubation [13]. The relative luciferase activity of the reporter containing the 3′ UTR of the *Y2R* gene was decreased by approximately 45% (Figure 4A), while the reporter containing the 3′ UTR of the *FGF18* and *SK* genes were not affected (Figure S3). These results suggested that *Y2R* could be regulated by Pm-miR-29a. Meanwhile, the expression of the *Y2R* gene in the Pm-miR-29a mimic-injected group decreased by 77.1% and 77.6% in the mantle and gill, respectively (Figure 4B,C), compared with the control group. Furthermore, *IL-17*, which was one pro-inflammatory factor, was upregulated 8.13- and 5.51-fold in the mantle and gill (Figure 5A), respectively. Moreover, the expression of *NF-κB* also increased 3.14- and 2.04-fold in the mantle and gill, respectively (Figure 5B). Meanwhile, we detected the expression of some nacre formation-related genes, including *nacrein*, *pearlin*, and *pif-177*, in the mantle tissues after Pm-miR-29a mimic injection. As expected, *nacrein*, *pearlin*, *pif-177* genes were all significantly inhibited, which gave further evidence of the effect of Pm-miR-29a on nacre-formation (Figure S4).

## 3. Discussion

miR-29a was initially discovered in humans and participated in a wide array of biological functions, such as immune response and bone formation [14,15]. Reports showed that mature miR-29a is highly conserved in the human, mouse, and rat [16]. Our results showed that the sequence of mature Pm-miR-29a was the same as that in cte-miR-29a, thereby indicating a relatively close genetic relationship. Despite slight differences among the sequences of different species, the analyzed mature miR-29a shared the same nucleotide at the seed region, played a key role in determining which protein-coding genes the miRNA would target, and indicated that the function of miR-29a may be conserved in different species.

In mammals, miR-29 is considered as an “osteo-miR”, which is involved in bone formation by regulating osteoblast differentiation [6] or collagen accumulation [7]. In fish and mouse, miR-29a expression was relatively high in the brain, and expression was present in all analyzed tissues, including calcified tissue, femur, and skull [9]. In *P. martensii*, Pm-miR-29a was easily detected in the analyzed tissues, which had lower expressions in the mantle and gill than in the adductor muscle, gonad, foot, and hepatopancreas. The mantle in the pearl oyster is the calcified tissues responsible for shell formation. The presence of Pm-miR-29a in the mantle further supported our hypothesis that it may serve a function in shell formation. Notably, after Pm-miR-29a over-expression in *P. martensii*, growth of the nacre in the shell was disrupted and a honeycomb growth pattern was observed. This phenomenon indicated that Pm-miR-29a could regulate nacre formation. Based on the function of miR-29a in mammals, we proposed that the disrupted growth after Pm-miR-29a over-expression is possibly caused by the inhibited differentiation of the calcified cells or by the reduced expression of some genes related to biomineralization.

We attempted to identify the target genes of Pm-miR-29a to elucidate the mechanism of shell formation. Y2R, a G protein-coupled receptor of neuropeptide Y (NPY), was predicted and experimentally proven as the target gene of Pm-miR-29a. Y2R is not only associated with bone regeneration, reconstruction and repair, but is also involved in the nerve immunity system, which plays a crucial role in regulating the inflammatory reaction and maintaining body homeostasis. The role of Y2R in bone formation and neuroimmunomodulation shows that the nervous system, the immune system, and biomineralization do not exist independently. Rather, they influence and interact with each other. Meanwhile, NPY is a potent anti-inflammatory peptide that directly modulates the activity of immune-related cells, such as granulocytes and macrophages, via its receptors [17]. NPY reportedly prevents nuclear translocation of NF-κB and inhibits interleukin 1β (IL-1β) release by activating Y1 receptors [18]. IL-17 is the only interleukin identified in *P. martensii* (PfIL-17) that exhibits potential function in immune response against extracellular pathogens by activating the NF-κB signal pathway [19]. Our analysis showed that Pm-miR-29a over-expression could increase the expression of *PfIL-17* and *NF-κB*, indicating that Pm-miR-29a could activate the *P. martensii* immune response against intracellular pathogens.

In mammals, miR-29a was also reported to play a dominant role in modulating several kinds of diseases and in the immune response of the organism. However, over-expression of miR-29a suppressed the immune responses induced by intracellular pathogens by targeting IFN-γ [20] and decreasing IL-6 [21] and TNF-α [21] secretions. The different functions of miR-29a in the immune response of mammals and *P. martensii* may be induced by the different target genes, thereby suggesting that the function of miRNA may change during evolution. Considering the reported interaction between immune system and biomineralization in vertebrates, we speculated that the function of pm-miR-29 in biomineralization may be mediated by the immune response induced by the activated *IL-17* and *NF-κB*. Furthermore, our analysis also demonstrated that some nacre formation-related genes, containing *pif-177*, *pearlin*, and *nacrein*, were significantly downregulated after over-expression of Pm-miR-29a, which elucidated the potential mechanism underling Pm-miR-29a in nacre formation. As no direct evidence was found to demonstrate the target interaction between Pm-miR-29a and these nacre formation-related genes, we proposed that the downregulation of these genes was not induced directly by Pm-miR-29a and may be mediated by other down-stream factors after *Y2R* inhibition or other regulatory target genes of Pm-miR-29a, which we have not found. Anyway, our results have provided the initial and final regulatory genes of Pm-miR-29a, while the detailed pathway in these processes was unclear until now, and it will be our next topic of research.

In conclusion, we verified the sequence of Pm-miR-29a by miR-RACE technology, and detected its expression level in various tissues of the pearl oyster *P. martensii*. Its function in nacre formation was characterized by over-expression of Pm-miR-29a *in vivo*. Furthermore, *Y2R* was identified as the potential target gene of Pm-miR-29a. We found that *IL-17* and *NF-κB* expression levels were upregulated, while nacre formation-related genes were downregulated after over-expression of Pm-miR-29a. Our results suggested that Pm-miR-29a participates in nacre formation and the immune response of *P. martensii* by targeting *Y2R*.

## 4. Experimental Section

### 4.1. Experimental Material

Adult pearl oysters *P. martensii* (about two years old) in the experiments were obtained from Liushawan, Zhanjiang, Guangdong Province, China, and preconditioned for 2 days at 25–30 °C in cistern with circulating seawater before use. All tissues, including adductor muscle, hepatopancreas, hemocytes, mantle, gonads, gills, and foot, were collected and sampled for RNA extraction.

### 4.2. Small RNA Extraction and Template Preparation

Small RNA was extracted from the mantle tissues by using RNAiso for Small RNA (Takara, Dalian, China), referring to the manufacturer’s instructions. Small RNA integrity was detected through 1% agarose gel electrophoresis, and purity was measured with A260/A280 ratio using NanoDrop 2000 Spectrophotometer (Thermo, Waltham, CA, USA). Subsequently, Poly A tail was added at the 3′ end of small RNA by Poly A Polymerase, and on this basis, a 5′ adaptor was connected through T4 RNA Ligase. About 500 ng small RNA was used for reverse transcription (RT) reaction with M-MLV reverse transcriptase (Promega, Madison, WI, USA) and universal RT-primer of miR-RACE. The related sequences used in this study are listed in Table 1.

**Table 1:** Primer sequences used in this study.

Primer Name	Primer Sequence	Function
5′ adaptor	CGACUGGAGCACGAGGACACUGAAAA	miR-RACE
GSP1	TTTTTTAAACTGATTTCAAATGGTGC	miR-RACE
mirRacer 5′ primer	CTGGAGCACGAGGACACTGA	miR-RACE
GSP2	CTGAAAATAGCACCATTTGAAATCA	miR-RACE
mirRacer 3′primer	ATTCTAGAGGCCGAGGCGGCCGACATG	miR-RACE
RT-primer	ATTCTAGAGGCCGAGGCGGCCGACATGTTTTTTTTTTTTTTTTTTTTTTT	miR-RACE
Pm-miR-29a (F)	TAGCACCATTTGAAATCAGTTT	qRT-PCR
Pm-miR-29a (R)	TGCGTGTCGTGGAGTC	qRT-PCR
Pm-miR-29a (RT)	GTCGTATCCAGTGCGTGTCGTGGAGTCGGCAATTGCACTGGATACGACAAACTGAT	qRT-PCR
*U6* (F)	ATTGGAACGATACAGAGAAGATT	qRT-PCR
*U6* (R)	ATTTGCGTGTCATCCTTGC	qRT-PCR
*U6* (RT)	ATTTGCGTGTCATCCTTGC	qRT-PCR
*GAPDH* (F)	CACTCGCCAAGATAATCAACG	qRT-PCR
*GAPDH* (R)	CCATTCCTGTCAACTTCCCAT	qRT-PCR
*Y2R*-F1	CGGACTAGTTCAAATATTCGATCGTGGGGAGCGTG	Vector Constructs
*Y2R*-R1	CCAAGCTTCGTGATGAGCCGATGACCTCTCTTGA	Vector Constructs
*FGF18*-F	CGGACTAGTTTCGGCACAGACGGGTAACATTTCC	Vector Constructs
*FGF18*-R	CCCAAGCTTTACTGGCCATGGGATCCTCGGTGT	Vector Constructs
*SK*-F	CGGACTAGTGTGATGAAAAATGCAAATCAGGGTC	Vector Constructs
*SK*-R	CCCAAGCTTAAATGCCATGTCGGAATTCAGTATATAC	Vector Constructs
*Y2R*-F2	TAGGGAGAACTTTAGCGGTCAA	qRT-PCR
*Y2R*-R2	AAATCCAATCGCAATGAGACC	qRT-PCR
*NF-κB*-F	AGAAGAGACAGGCCAAAGAGCA	qRT-PCR
*NF-κB*-R	AGAGAGAACAGGCGTGAGAAGC	qRT-PCR
*IL-17*-F	AAGAAAACTTTGAACATGCCGTAC	qRT-PCR
*IL-17*-R	TAATCACATAATGCCAGGGACA	qRT-PCR

GSP: Gene-Specific Primer; Pm: Pinctada martensii; RT: Reverse Transcription; *Y2R*: neuropeptide Y receptor type 2; *FGF18*: Fibroblast Growth Factor-18; *SK*: protein serine kinase; *IL-17*: interleukin 17; *NF-κB*: nuclear factor κB.

### 4.3. miR-RACE

miR-RACE was applied to further verify the precise sequences of Pm-miR-29a. The gene-specific primers (GSP) for miR-RACE were designed based on the sequence of Pm-miR-29a, which was obtained by deep sequencing in our previous research. Then 5′ miR-RACE was performed using mirRacer 5′ primer and Pm-miR-29a gene-specific forward primer (GSP1); similarly, 3′ miR-RACE amplification was performed using mirRacer 3′ primer and Pm-miR-29a gene-specific reverse primer (GSP2) [22]. All primer sequences are listed in Table 1. The PCR products with a single band were ligated to pMD-18T vector (Takara) and then transformed into competent DH5a cells, and the positive monoclones to be sequenced were then selected.

### 4.4. Quantitative Real-Time PCR (qRT-PCR)

According to the manufacturer’s instructions, total RNA was extracted from all stored tissues using Trizol reagent (Invitrogen, Carlsbad, CA, USA); these RNA served as templates for the synthesis of cDNA first chain. We then used the precise sequences of Pm-miR-29a as the forward primer and universal primer as the reverse primer to amplify the Pm-miR-29a from the reverse-transcribed cDNA (Table 1) [23]. Primers for *Y2R*, *NF-κB* and *IL-17* were listed in Table 1. qRT-PCR was advanced by Applied Biosystems 7500/7500 Fast real-time system (ABI, Foster City, CA, USA). Each sample was run in triplicate along with *U6 snRNA* or *GAPDH* as the reference gene.

### 4.5. Target Gene Prediction

The reported genes from *P. martensii* were downloaded from NCBI GenBank (http://www.ncbi.nlm.nih.gov/nuccore/). RNAhybrid software was used to predict the target genes of Pm-miR-29a. Based on the gene annotation and target prediction results, the potential targets of Pm-miR-29a were screened as follows: (1) the calculated free energy in the hybridization site was lower than −20 kcal/mol; and (2) information on gene annotation was related to the immune response or biomineralization, which is the reported function of miR-29a in vertebrates [9,20].

### 4.6. Over-Expression of Pm-miR-29a in Vivo

Pm-miR-29a and Negative Control mimics were synthesized by Genepharma, Shanghai, China, and were diluted in 0.1 μg/μL with RNase-free water. Subsequently, about 100 μL solutions were injected into the adductor muscle of different individuals separately, and the same doses were injected again after four days. In this experiment, 10 individuals were used in each group. At 8-day after the first injection, the mantle and gill were sampled for total RNA extraction to be used in stem loop qRT-PCR analysis. qRT-PCR was carried out to detect the expression levels of Pm-miR-29a and nacre formation-related genes or the putative target genes. Meanwhile, the collected shells in each group were cut into pieces (about 1 cm × 1 cm), rinsed with purified water, and dried. At least three pieces of shells were coated with gold and observed under scanning electron microscope (SEM) (JEOL, Tokyo, Japan) [24].

### 4.7. Vector Construction

The 3′ UTR of the selected target genes containing miRNA-binding sequences was amplified using the listed primers in Table 1. The purified PCR products were subcloned into the SpeI/HindIII site in the pMIR-REPORT luciferase reporter vector (Ambion, Waltham, CA, USA). Whether the vector construction was successful or not was determined by sequencing.

### 4.8. Cell Culture and Transfection

HEK-293T cell was cultured at 37 °C in Dulbecco’s Modified Eagle’s Medium (DMEM) high glucose containing 10% fetal bovine serum in a CO_2_ incubator with 5% CO_2_ to ensure a saturated humidity condition for the cells. Transfection of plasmid was performed in strict accordance with the manufacturer’s instructions of Lipofectamine™ 2000 (Invitrogen, Carlsbad, CA, USA). The day before transfection, HEK-293T cells in logarithmic phase were seeded into a 48-well culture plate, and the number of inoculated cells was 5 × 10^4^ per hole with 500 μL medium. Thus, the density of the transfected cells could reach 70%–80% the next day. Before transfection, the medium was replaced with a fresh one. Meanwhile, the constructed plasmid was co-transfected with Pm-miR-29a or Negative Control mimics; pMIR-REPORT luciferase empty vector was used as the control. Furthermore, each well was transfected with 4 ng plasmid pRL-TK vector as an internal quality control to correct the differences among the groups. At 48 h after the transfection, the cells were lysed, and the luciferase activity was tested using dual-luciferase assay kit (Promega, Fitchburg, WI, USA).

### 4.9. Statistical Analysis

All data are presented as mean ± SD. SPSS software (IBM, Chicago, IL, USA) was used for the statistical analysis in a one-way ANOVA. A difference with *p* < 0.05 was deemed to be statistically significant by Duncan’s multiple comparison test.

## Supplementary Materials

Supplementary materials can be found at http://www.mdpi.com/1422-0067/16/12/26182/s1.
