# Supplementary material for: miR-29a Participated in Nacre Formation and Immune Response by Targeting Y2R in Pinctada martensii
Source: Int J Mol Sci. 2015 Dec 10;16(12):29436–45. doi: 10.3390/ijms161226182 (PMC4691125; doi:10.3390/ijms161226182)
Supplement: Supplementary file 1 [file ijms-16-26182-s001.pdf]

Supplementary Materials: miR-29a Participated in Nacre Formation and Immune Response by Targeting Y2R in *Pinctada martensii*

Rongrong Tian, Zhe Zheng, Ronglian Huang, Yu Jiao and Xiaodong Du

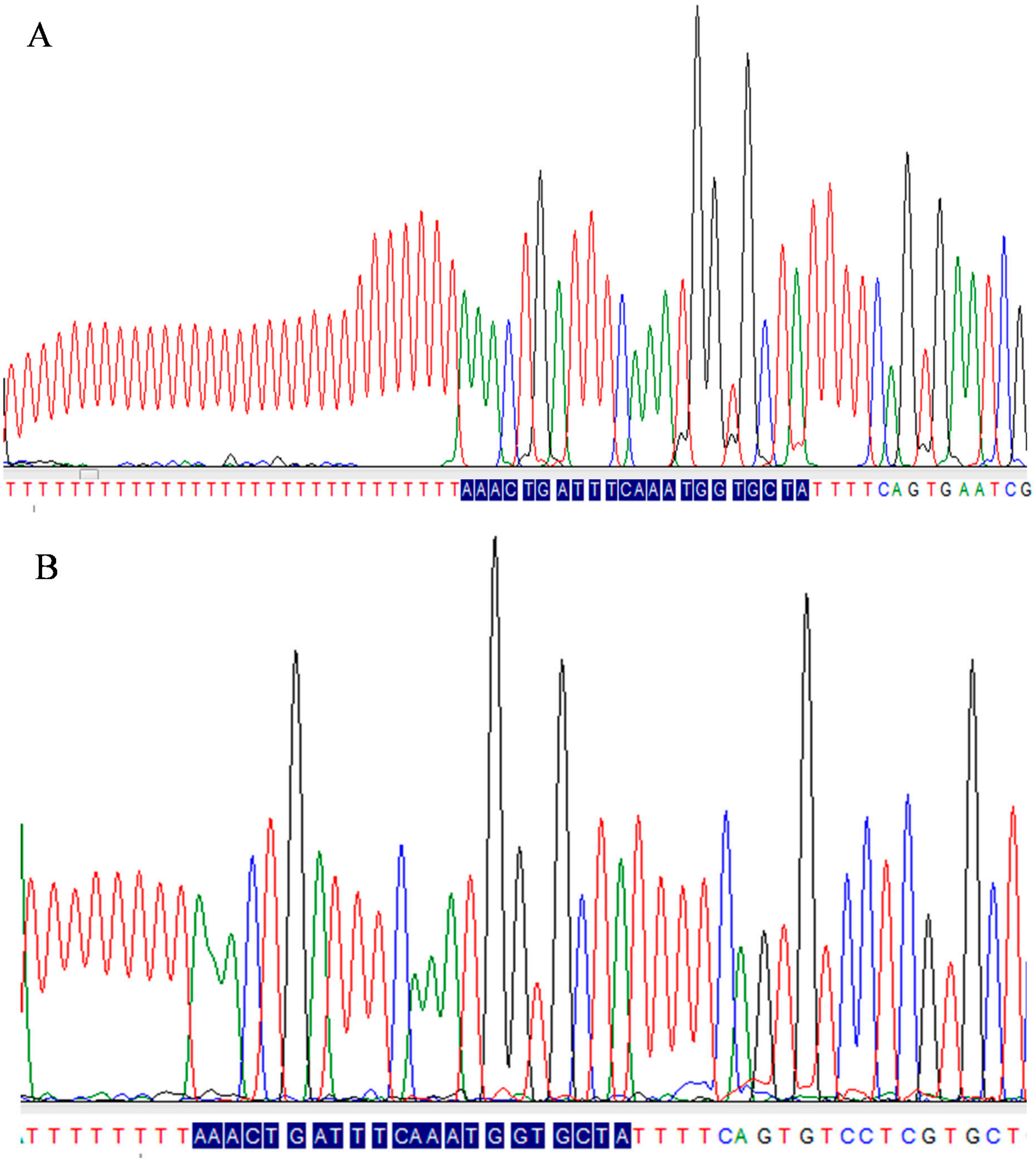

Figure S1. Sequence verification of the mature pm-miR-29a by miR-RACE. The result of 3′ miR-RACE (**A**) and 5′ miR-RACE (**B**), highlighted parts represented the mature sequence of pm-miR-29a.The different colors means different bases, red is T, green is A, blue is C and blank is G. The curves means the reliability of sequencing.

```
target: SOCS-2 (cytokine-induced suppressor of cytokine signaling 2)
miRNA: miR-29a
mfe: -18.2 kcal/mol
position  310
target 5’   U   UA    GU UC        U 3’
             UUG  GUUU  A  GUGGUGCU
             GAC  UAAA  U  UACCACGA
miRNA  3’ UUU         GU           U 5’
```

```
target: toll-like receptor 3
miRNA: miR-29a
mfe: -14.3 kcal/mol
position  159
target 5’ A         UGUUUGUUUC     AA   A    3’
           AAAUUGGUU          UCAAG  GGU
           UUUGACUAA          AGUUU  CCA
miRNA  3’                          A    CGAU 5’
```

```
target: FGF18(fibroblast growth factor18)
miRNA: miR-29a
mfe: -24.0 kcal/mol
position  871
target 5’  U            AAG       U 3’
            AUUG UUUCAAA   GGUGCUA
            UGAC AAAGUUU   CCACGAU
miRNA  3’ UU    U       A           5’
```

```
target : TNF-alpha factor
miRNA: miR-29a
mfe: -18.6 kcal/mol
position  181
target 5’    A   AA   UA       U 3’
              UGA  UCA   GGUGCU
              ACU  AGU   CCACGA
miRNA  3’ UUUG   AA   UUA      U 5’
```

```
target: N19 (Nacre protein)
miRNA: miR-29a
mfe: -18.6 kcal/mol
position  285
target 5’    A   GGAAAA    U    AG    A 3’
              UGA      UUCA AUGG  GCUG
              ACU      AAGU UACC  CGAU
miRNA  3’ UUUG   A         U    A       5’
```

```
target: alkaline phosphatase
miRNA: miR-29a
mfe: -15.8 kcal/mol
position  119
target 5’ C     CCG           A   3’
           AAUUG   UUA   GGUGC
           UUGAC   AGU   CCACG
miRNA  3’ U     UAA   UUA     AU 5’
```

```
target: IRF-2 (interferon regulatory factor 2)
miRNA  : miR-29a
mfe: -18.4 kcal/mol
position  122
target 5’ U       GUU      CAAUUGA  A   3’
           GACUGAU    GAUGG       GC
           UUGACUA    UUACC       CG
miRNA  3’ U       AAGU     A        AU 5’
```

```
target: dermatopontin
miRNA  : miR-29a
mfe: -11.8 kcal/mol
position  103
target 5’  U   AA              C   3’
              ACU  AUUUCA    GUGU
              UGA  UAAAGU    CACG
miRNA  3’ UU   C       UUAC    AU 5’
```

```
target:IL-17( interleukin 17 )
miRNA  : miR-29a
mfe: -13.9 kcal/mol
position  37
target 5’ U     UG       UUA  C    C 3’
           GGCUG    CAGAU   GU GUUG
           UUGAC    GUUUA   CA CGAU
miRNA  3’ U     UAAA     C           5’
```

```
target: SK (protein serine kinase)
miRNA: miR-29a
mfe: -20.1 kcal/mol
position  71
target 5’    A  UUAUAG    U        U 3’
              UG      UCAA GUGGUGCU
              AC      AGUU UACCACGA
miRNA  3’ UUUG  UAA                U 5’
```

```
target: allograft inflammatory factor-1
miRNA: miR-29a
mfe: -15.8 kcal/mol
position  308
target 5’ C       CAGGCAACA       CU      A 3’
           AAACUGA         UUUUAAA   UGUUA
           UUUGACU         AAAGUUU   ACGAU
miRNA  3’                         ACC       5’
```

```
target: amorphous calcium carbonate binding protein 1
miRNA: miR-29a
mfe: -14.2 kcal/mol
position  119
target 5’        A      A  CAAG     A 3’
                  UUCAAA GG    UGUUA
                  AAGUUU CC    ACGAU
miRNA  3’ UUUGACUA      A             5’
```

```
target: Y2R(neuropeptide Y receptor type 2)
miRNA: miR-29a
mfe: -20.6 kcal/mol
position  365

target 5’ A        AAAUUUG   C         G 3’
           GAACUGAU       CAG AUGGU CUA
           UUUGACUA       GUU UACCA GAU
miRNA  3’          AA              C     5’
```

```
target: PfTy (tyrosinase-like protein)
miRNA: miR-29a
mfe: -17.6 kcal/mol
position  157
target 5’   U   CAAGA     U     AG    C 3’
               CUG     GUUUC  GUGG  GCUA
               GAC     UAAAG  UACC  CGAU
miRNA  3’ UUU             UU    A       5’
```

```
target:BMP-7(bone morphogenetic protein 7)
miRNA: miR-29a
mfe: -18.9 kcal/mol
position  1099
target 5’   U        UU   UCUUAUAUGUUGCAA      U 3’
             UUGAUUUU  AUG               GUGCUA
             GACUAAAG  UAC               CACGAU
miRNA  3’ UUU        UU                          5’
```

```
target: tissue inhibitor of matrix metalloproteinase
miRNA: miR-29a
mfe: -12.4 kcal/mol
position  124
target 5’ A    GCCAAG          A    3’
           AAAU      UUCA  UGGU
           UUUG      AAGU  ACCA
miRNA  3’      ACUA      UU    CGAU 5’
```

Figure S2. Target prediction by RNAhybrid. RNAhybrid software was used to predict the target relationship between pm-miR-29a and the 3′ UTR of known genes which was related to the immune response or biomineralization in *P. martensii*.

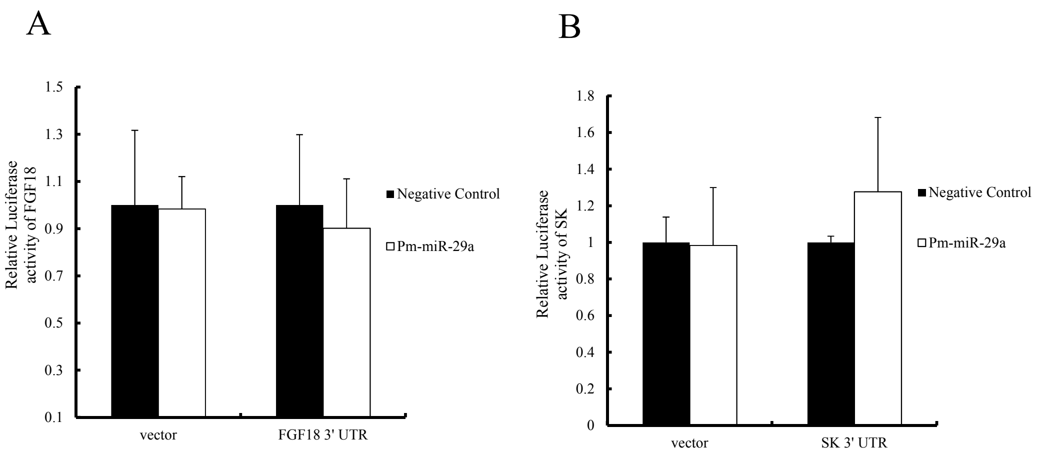

Figure S3. The relative luciferase activity of the reporter containing the 3′ UTR of *FGF18* and serine kinase (*SK*) gene. The luciferase activity of the reporter containing the 3′ UTR of *FGF18* (**A**) and *SK* (**B**) gene were not affected by pm-miR-29a mimics.

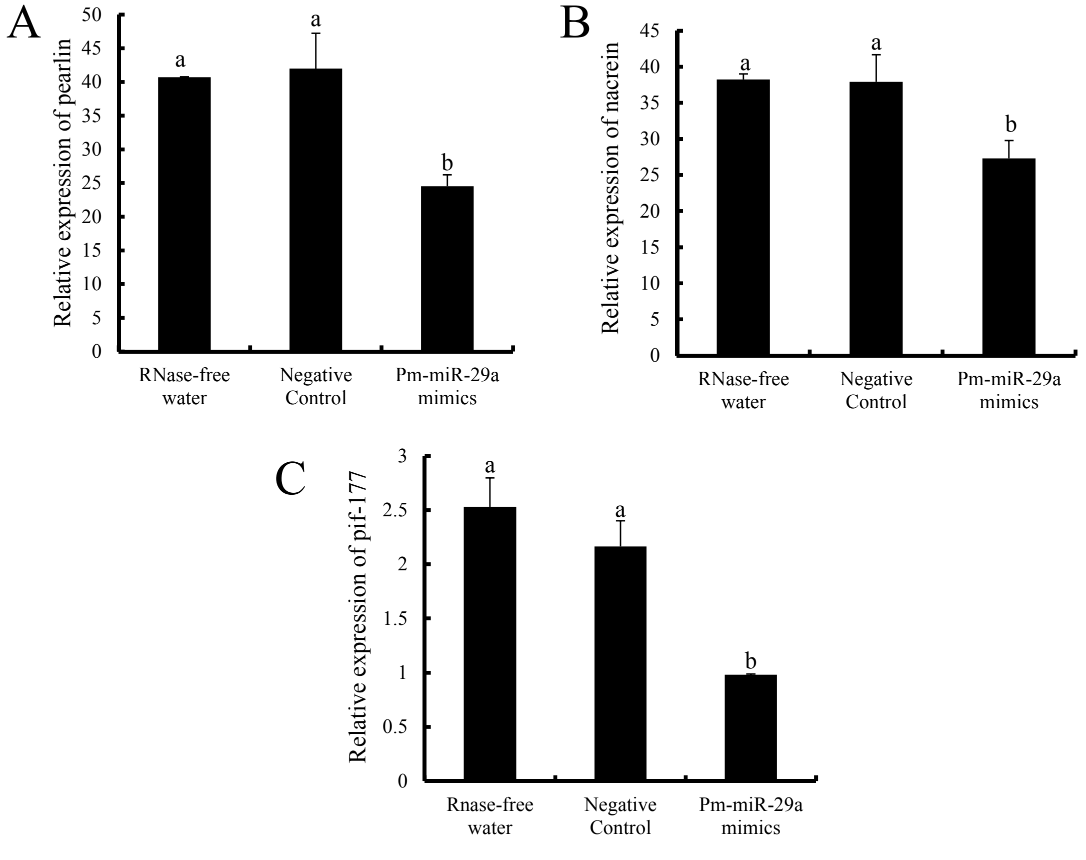

Figure S4. Expression of nacre formation-related genes after over-expression of pm-miR-29a. qRT-PCR was applied to detect the expression of nacre formation-related genes including *pearlin*, *nacrein* and *pif-177* in the mantle tissues of five individuals after pm-miR-29a mimics injection. Results showed that the expression of *pearlin* (**A**), *nacrein* (**B**), *pif-177* (**C**) were significantly down-regulated after over-expression of pm-miR-29a. Different letters mean a significant difference ($p < 0.05$). Error bars correspond to mean ± SD.
